# Is *CABP2*‐Associated Hearing Loss (DFNB93) a Gene Therapy Target? Preclinical Progress and Patient Registry

**DOI:** 10.1002/mco2.70363

**Published:** 2025-09-08

**Authors:** Barbara Vona, Bernd Wollnik, Nicola Strenzke, Tina Pangršič, Tobias Moser

**Affiliations:** ^1^ Institute of Human Genetics University Medical Center Göttingen Göttingen Germany; ^2^ Institute for Auditory Neuroscience and InnerEarLab University Medical Center Göttingen Göttingen Germany; ^3^ Auditory Neuroscience and Optogenetics Laboratory German Primate Center Göttingen Germany; ^4^ Collaborative Research Center 1690 (CRC1690) University of Göttingen Göttingen Germany; ^5^ Cluster of Excellence “Multiscale Bioimaging: from Molecular Machines to Networks of Excitable Cells” (MBExC) University of Göttingen Göttingen Germany; ^6^ Department of Otolaryngology University Medical Center Göttingen Göttingen Germany; ^7^ Auditory Neuroscience and Synaptic Nanophysiology Group Max Planck Institute for Multidisciplinary Sciences Göttingen Germany

**Keywords:** cabp2, calcium‐binding protein 2, DFNB93, hearing impairment, patient registry, synaptopathy

## Abstract

*CABP2* modulates presynaptic Ca_V_1.3 Ca^2+^ channel function in inner hair cells (IHCs) and is required for indefatigable synaptic sound encoding. Biallelic variants in *CABP2* are associated with non‐syndromic hearing loss (DFNB93). Otoacoustic emissions have been observed in an Italian family with a homozygous *CABP2* variant, indicating preservation of outer hair cell‐mediated cochlear amplification. Hence, DFNB93 belongs to the hearing disorders caused by impairment of IHC synapses, termed auditory synaptopathy. DFNB93 mouse models have recapitulated findings and demonstrated that lack of CaBP2 impairs synaptic sound encoding by enhanced steady‐state inactivation of Ca_V_1.3 Ca^2+^ channels. Furthermore, preclinical studies have demonstrated feasibility of gene therapy. As growing evidence from *OTOF* clinical trials confirms synaptopathies as promising therapeutic targets for hearing restoration, *CABP2* ranks highly among the candidate genes for virus‐mediated gene therapy to restore hearing. This perspective summarizes the preclinical gene replacement studies for hereditary hearing loss and outlines the characteristics that make genetic targets ideal for therapy development. It reviews the current literature on human *CABP2* studies, pre‐clinical therapy development, and introduces a patient registry that aims to support research involvement with the *CABP2* patient community. We conclude with a preview of the next steps toward *CABP2* gene therapy clinical trials.

## Graphical abstract

The intended aims and benefits of the *CABP2* Patient Registry.

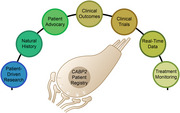

## 1 Introduction

Hearing impairment is the most common sensory disorder in humans, affecting approximately 432 million adults and 34 million children to an extent that poses significant challenges for communication and daily life, with more than 700 million people projected to be affected by 2050 [1]. In the Western world, roughly one to two per 1000 newborns are diagnosed with hearing impairment, with at least 60% of cases attributed to genetic causes [2, 3]. The prevalence of hearing impairment increases dramatically with age, affecting over 80% of individuals by the age of 80 years [4]. In children, hearing impairment can adversely impact speech and language development, as well as education, while in adults, it has been linked to depression and cognitive decline, all of which impact quality of life. Beyond the critical period of speech and language development, hearing deficiency can contribute to difficulties in maintaining relationships that are helpful for emotional resilience and social well‐being throughout life.

The experiences of individuals with hearing impairment and deafness are diverse, covering a range of perspectives, identities, and values. Many individuals identify as part of the Deaf community, which embraces deafness as a cultural and linguistic identity rather than a deficit or disability [7]. A respectful and inclusive treatment that acknowledges the linguistic and cultural identities and personal journeys is important in healthcare for deaf individuals [8]. This includes the support of informed choice for patients regarding genetic testing, therapeutic interventions, and involvement in research questions. Our registry is meant to serve as a resource for researchers and patients to interact and exchange knowledge and data.

Despite the importance of hearing, major barriers to accessing multidisciplinary hearing healthcare persist for those seeking therapeutic options. This also applies to higher income countries where hearing healthcare may be partially or fully covered by national healthcare schemes, as cost and stigma remain common obstacles to access [9]. Early clinical diagnosis and intervention can support access to communication tools and resources for helping individuals more fully engage with their environments. Regardless of its underlying cause, hearing impairment poses a major global challenge that demands multifaceted solutions for those seeking solutions. Standard treatment options for hearing restoration, irrespective of the exact disease mechanism, include hearing aids, which amplify sounds for individuals with mild to moderate hearing impairment, and electrical cochlear implants for individuals who do not sufficiently benefit from acoustic amplification. Patients with auditory synaptopathy and auditory neuropathy typically gain little benefit from using hearing aids, as the functional problem is not hearing sensitivity but neural sound processing. The outcome of cochlear implant rehabilitation depends on several factors, such as the neural status of the cochlea, and is limited by poor spectral sensitivity [10, 11]. Current developments include improved neural‐electrode interfacing and optogenetic cochlear implants for better spectral selectivity, as well as regenerative strategies for improving the neural status of the cochlea [10, 11]. Moreover, causative treatment options, such as by gene replacement, in monogenic deafness are emerging (Table 1). Genetic auditory synaptopathy is an attractive target for gene therapy as the structure and integrity of the cochlea are relatively well preserved. Encouraging signs come from the early stages of the first clinical trial for otoferlin‐associated hearing impairment, setting a blueprint for further clinical trials [12, 13, 14]. Genetic testing has become an important routine tool for hearing healthcare in individuals with unlikely environmental or non‐genetic causes. These developments highlight a growing momentum toward precision medicine in the treatment of hearing impairment, where understanding the underlying genetic causes can lead to more effective individualized therapies. A detailed understanding of the exact disease mechanism is crucial for optimal clinical counseling of patients with hearing impairment and specifically in those with auditory synaptopathy. As gene‐based interventions continue to emerge, patient registries are set to become essential tools for connecting patients with research efforts to support future clinical trials.

**TABLE 1:** Preclinical gene replacement approaches in mouse models of hereditary deafness.

Gene therapy approach															Phenotyping									References
	Target cells^a^							Delivery									Time of (first) testing	Tone burst ABR threshold^b^ dB (SPL)			Click ABR threshold dB (pe)			
Gene	IHC	OHC	SGN	SC	SV	Injection age	Vector	RWM	Utricle	Cochleostomy	Scala media	Canalostomy	Transuter. otocyst	Superficial temporal vein	Longitudinal study	Vestibular phenotyping	Mouse model (if more than one relevant)	Treated mutant	Untreated mutant	WT‐like control	Treated mutant	Untreated mutant	WT‐like control	Reference
*Cabp2*	x					P5–P7	AAV2/1	x							—	—	4–7 weeks following injection	55	—	40	60	—	45	[15]
*Cabp2*	x					P5–P7	AAV‐PHP.eB	x							—	—	4–7 weeks following injection	55	70	35	40	55	30	[15]
*Cabp2*	x					P6	AAV‐PHP.eB	x							—	—	2–3 weeks after injection	50	85	35	40	60	30	[16]
*Cdh23*		x				4–5 weeks	Triple‐AAV2/2	x							+	—	Age: 12–60 weeks	No prevention of age‐related hearing deterioration						[17]
*Clrn1*	x	x	x			P1–P3	AAV2, AAV8	x							+	—	Age: 4–22 weeks (Ko‐TgAC1 mouse model)	—	—	—	45	45–85	30	[18]
*Clrn1*	x	x	x			P2–P3	AAV2/8	x								—	Age 4 weeks (*Clrn1^ex4−/−^*)	90	105	20	—	—	—	[19]
															2 months		Age 4 weeks (*Clrn1^ex4fl/fl^ Myo15‐Cre* ^+/−^)	25	70	20				
*Clrn1*	x	x	x			P1	AAV9‐PHP.B	x								—	4 weeks post‐injection	85	95	30				[20]
*Clrn1*	x	x	x			P1	AAV‐S	x							Up to 5 months	—	Age 5 weeks (Ko‐TgAC1 mouse model)	30	80	30	—	—	—	[21]
*Clrn2*	x	x				P1 (P5–P14)	AAV9‐PHP.eB	x							Up to 6 months	—	Age 4 weeks (thr. for P1 injection)	35	90	25	—	—	—	[22]
*Eps8*	x	x				P1–P2	Anc80L65	x							—	—	Age 3–5 weeks	No hearing improvement						[23]
*Gjb2*				x		P0–P1	AAV2/1			x	x				—	—	Age 1 month	No hearing improvement						[24]
*Gjb2*				x		P0 (P42)	AAV1	x							—	—	Age 2–3 months (thr. for P0 injection)	75	90	—	—	100	30	[25]
*Gjb2*				x		P28	Anc80	x							—	—	1 week–1 month after injection	No hearing improvement						[26]
*Gjb2*				x		P0, P1	AAV9‐PHP.B‐GRE	x							Up to 2 months	—	1 and 2 month(s) post‐injection (*Gjb2^Pfl/Pfl^, Cre^+^*)	105	120	50	120	125	55	[27]
																	(*Gjb2^Gjb6−^*)	35	110	35	60	120	60	
*Gjb2*				x		P3–P4 (P14, P30)	Co‐administration of AAV1 and AAV‐ie	x							Up to 6 months	—	1 month post‐injection at P4 *(Sox9^CreER/+^/Gjb2^loxP/loxP^)*	45	75	—	60	80	—	[28]
															Up to 3 months		1 month post‐injection at P3–P4 *(P0^Cre^/Gjb2^loxP/loxP^)*	45	90	—	60	90	—	
*Gjb6*				x		E11.5	Plasmid electroporation						x		—	—	Age P30	30	75	25	—	—	—	[29]
*Gjb6*				x		P4	Bovine AAV					x			—	—	4 weeks post‐injection	No hearing improvement						[30]
*Gjb6*				x		P0–P2	AAV1 (*Gjb2* construct)				x				Up to 3 months	—	1 month post‐injection	45	75	25	50	90	35	[31]
*Ildr1*	x	x		x	x	P0–P5	Dual AAV2.7m8/AAV8BP2					x			Up to 2 months	—	2 weeks post‐injection	60	75	30	—	—	—	[32]
*Kcne1*					x	P0–P2	AAV1					x			Up to 6 months	Rescue	30 days after injection	55	> 100	20	—	—	—	[33]
*Kcnq1*					x	P0–P2	AAV1				x				Up to 30 weeks		4 weeks after injection	50	90	30	45	> 90	30	[34]
*Lhfpl5*	x	x	x			P0–P2	Exo‐AAV1	x		x					—	Rescue	4 weeks after P1 RMW injection (nine out of 12 animals)	80	> 100	30	—	—	—	[35]
*Msrb3*	x	x				E12.5	rAAV						x		Up to 7 weeks	—	Age 4 weeks	25	100	20	25	100	20	[36]
*Myo7a*	x	x				P0–P5	Dual AAV8					x			—	Rescue	Age approx. P30	No hearing improvement						[37]
*Myo7a*	x	x				P16 (P4)	Lentivirus					x			—	Rescue	Age 3 months (ABR improv. for P16, not P4 injection) (Shaker‐1*^mut^*)	75	100	40	—	—	—	[38]
																—	Age 6 months for P4 injection (Shaker‐1*^het^*)	40	65	40				
*Ndp*			x			P2, P21–P30	AAV9							x	—	—	Age 3 months for (thr. for P2 injection) lower efficacy with later injections	35	65	35	30	50	30	[39]
*Otof*	x					P6–P7	Dual AAV2/6	x							—	—	Age 4 weeks	70	> 100	30	50	> 110	30	[40]
*Otof*	x					P10, P17, or P30	Dual AAV2	x							Up to 7 months	—	Thr. for P30 injection, 3 weeks after injection	50	>90	50	35	>90	35	[41]
*Otof*	x					P5–P7	Single AAV‐PHP.eB (overload)	x							Up to 6 months	—	Age 5 weeks (∼40%–70% of animals)	60	> 100	—	60	95	—	[42]
*Otof*	x					P0–P2, P30	Dual AAV‐PHP.eB	x							Up to 6 months	—	1 month after injection at P0–P2	45	>90	40	45	>90	45	[43]
*Otof*	x					P0–P2, P30	Dual AAV1	x							Up to 6 months	+	2 weeks after injection at P30	65	>90	40	60	>90	40	[44]
*Otof*	x					P30	Dual AAV1/Dual Anc80L65	x							Up to 4 months	+	1 week post‐injection	40	90	35	60	90	40	[45]
*Otof*	x					P0–P2, P30	Dual AAV‐PHP.eB	x							Up to 6 months for P0 inj.	—	2 weeks after injection at P30	35	> 95	55	60	> 100	50	[46]
*Otof*	x					P0–P2	Dual AAV‐PHP.eB	x							Up to 52 weeks	—	4 weeks post‐injection (with mid‐Myo15 promoter)	45	>95	40	45	>95	40	[47]
*Pcdh15*	x	x				P1	AAV9‐PHP.B (mini Pcdh15)	x							—	—	Age 5 weeks *(Pcdh15^fl/fl^, Myo15a‐Cre^+/−^)*	45	125	30	55	125	50	[48]
																	Age 5 weeks *(Pcdh15^−/−^)*	90	125	30	—	—	—	
*Pcdh15*	x	x				P1	Dual AAV‐ AAV9‐PHP.B	x							—	—	Age 5 weeks *(Pcdh15^fl/fl^, Myo15a‐Cre^+/−^)*	50	85	30	60	85	50	[49]
															—	Rescue	Age 5 weeks *(Pcdh15^−/−^)*	—	—	—	—	—	—	
*Pjvk*	x	x	x			P3	AAV2/8	x							—	—	Age 3 weeks	55	85	—	—	—	—	[50]
*Pjvk*	x	x	x			P0–P1	Anc80L65	x							Up to 2 months	Rescue	Age 3 weeks	50	90	—	55	95	—	[51]
*Slc26a4*					x	E12.5	rAAV2/1						x		Up to 3 months	No rescue	Age 3–5 weeks (both mouse models)	40	>90	20	25	>90	15	[52]
*Slc26a4*					x	E11.5	Plasmid electroporation						x		—	Rescue	P30	50	85	35	—	—	—	[53]
*Slc26a5*		x				P2	AAV‐ie	x							—	—	Age 4 weeks	70	90	25	—	—	—	[54]
*Slc26a5*		x				P3, P30	AAV‐ie‐B8 (P3) AAV2‐B8‐nls‐mNeonGreen (P30)	x							—	—	P3 transduction, P30 ABR; P30 transduction, P40 ABR	30	80	30	—	—	—	[55]
*Strc*		x				P1	Dual AAV9‐PHP.B		x						—	—	Age 4 weeks restored DPOAE w/o DPOAE (∼50% of animals)	50 85	85	25	—	—	—	[56]
*Syne4*		x				P0–P1.5	AAV9‐PHP.B					x			Up to 12 weeks	—	Age 4 weeks	20	70	10	—	—	—	[57]
*Tmc1*	x	x				P0–P2	AAV2/1	x							—	—	P25 to P30 post‐injection	90	> 115	—	—	—	—	[58]
*Tmc1*	x	x				P1–P2 P4, P7, P14	sAAV (AAV2/Anc80L65)	x							Up to 3 months (P1 inj.)	Rescue	Age 4 weeks (thr. for P1–P2 injection) lower efficacy with later injections	55	> 115	30	—	—	—	[59]
*Tmc1*	x	x				P1 (P7)	AAV9‐PHP.B		x						Up to 12 weeks	—	4 weeks post‐injection (thr. for P1 inection)	60	110	20	—	—	—	[60]
*Tmc1*	x	x				P1–P2	AAV9‐PHP.B		x						Up to 8 weeks	—	Age 4 weeks (*Tmc1^∆/∆^*)	50	110	30	—	—	—	[61]
															Up to 12 weeks	—	Age 4 weeks (*Tmc1^N193I/N193I^*)	35	110	25	—	—	—	
*Tmprss3*	x	x	x	x		P1	AAV‐KP1/AAV‐DJ					x			Up to 4 months	—	Age 3 weeks	65	>80	40	—	—	—	[62]
*Tmprss3*	x	x	x	x		18.5 months	AAV2	x							Up to 5 months post inj.	—	1 month post‐injection (age 19.5 months)	40	50	35	—	—	—	[63]
*Ush1c*	x	x				P0–P1	AAV2/Anc80L65	x							Up to 6 months	Rescue	Age 6 weeks	45	> 110	25	—	—	—	[64]
*Ush1g*	x	x				P2.5	AAV8	x							Up to 12 weeks	Rescue	Age 4 weeks	75	> 110	30	—	—	—	[65]
*Ush1g*	x	x				P12–P21 (P22–P30)	AAV2/Anc80L65	x							Up to 2 months	Rescue	P40 (hearing restoration with earlier injection only)	85	120	20	—	—	—	[66]
*Vglut3* ^c^	x					P1–P3, P10–P12	AAV1	x		x					Up to 1.5 years	—	40 days post‐delivery	60	90	55	40	90	30	[67]
*Vglut3* ^c^	x					P3, P5, P8 (20 weeks)	AAV8					x			Up to 21 weeks	+	1 week post‐injection (thr. for 5 weeks injection)	25	>90	20	—	—	—	[68]
*Whrn*	x	x				P1–P5	AAV8	x							—	—	Age 1–3 months	No hearing improvement						[69]
*Whrn*	x	x				P1–P5	AAV8					x			Up to 4 months	Rescue	P30 (8 kHz only, data from eight of 29 animals)	80	100	20	—	—	—	[70]

Abbreviations: ABR, auditory brainstem response; IHC, inner hair cell; OHC, outer hair cell; P, postnatal day; RWM, round window membrane; SC, supporting cell; SGN, spiral ganglion neuron; SPL, sound pressure level; thr, threshold; WT, wild‐type.

^a^ The majority of the studies used ubiquitous promoters, so expression was often observed elsewhere in addition to the target cells.

^b^ Mean tone‐burst ABR threshold is the average of the threshold at two neighboring frequencies with the best rescue result.

^c^ The official Human Gene Organization Gene Nomenclature Committee name for *Vglut3* is *SLC17A8*.

This article reviews preclinical gene replacement strategies for the treatment of hereditary hearing loss, touches on the criteria of a good therapeutic target, and outlines qualities of *CABP2* that make it an excellent therapeutic target. In anticipation of clinical trials, we developed and released a registry for *CABP2*‐associated hearing impairment. As we build on experience from the development of the Otoferlin Registry (Clinical Trials Identifier: NCT05946057 [71]), these tools are likely to play a crucial role in supporting research and translation, a topic that will be briefly explored here.

## 2 Preclinical Gene Replacement Strategies for Hereditary Hearing Loss

The human inner ear is a complex, fluid‐filled, three‐dimensional structure encased within the densest bone of the body and located deep in the base of the skull [72]. The remarkable complexity of the auditory system is mirrored by extensive genetic heterogeneity, with over 155 genes identified to date as causal for non‐syndromic (isolated) hearing impairment (https://hereditaryhearingloss.org/). Efforts of the last 15 years have revolutionized genetic sequencing technologies that make it possible to analyze all hearing loss‐associated genes in patients, expediting diagnostics and research [73]. Given the diversity of genetic causes, comprehensive molecular genetic testing has proven essential for determining the precise basis of hearing impairment, to define non‐syndromic versus syndromic forms, and is a crucial part of clinical trial eligibility among other benefits of a genetic diagnosis (summarized previously [3]).

To address genetic heterogeneity, the field has undertaken major efforts in preclinical development of gene therapies (reviewed previously [74, 75]), including gene replacement approaches that have been applied to mouse models to combat hearing loss caused by a variety genes, as summarized in Table 1. Gene replacement involves delivery of a coding gene sequence (transgene), along with regulatory elements, promoters, enhancers, and other stabilizing sequences in a transgene cassette, to the target cell with viral vectors, typically using adeno‐associated virus (AAV). AAVs have, so far, emerged as the vector of choice due to good safety profile, including low immunogenicity, sustained transgene expression, and modifiable capsids to tailor vectors in specific cell types [74, 75]. Gene replacement is also the approach applied in the recent otoferlin clinical trials (summarized previously [76]).

## 3 Criteria for Gene Therapeutic Targets for Hearing Restoration

A strong therapeutic candidate for gene therapy must meet several key criteria. First, the timing of cochlear degeneration must allow intervention before irreversible hair cell loss occurs, as human hair cells do not regenerate, known as “therapeutic window.” The course of hearing loss reported in human natural history studies combined with histological studies in mouse models have been important for estimating this therapeutic window. While the human inner ear starts to respond to sounds at ∼20 weeks gestation [77], hearing in the mouse starts roughly 2 weeks after birth (∼P12–P14). For a comparative timeline, genes associated with stable cochlear architecture and hair cell presence beyond the equivalent of the human postnatal period (e.g., P28 in mice) are ideal for therapy [78]. Second, the expression profile of the target gene must align with AAV tropism. Advances in capsid engineering of synthetic AAV variants (e.g., Anc80L65 and AAV9‐PHP.B) have enhanced transduction and therapeutic efficacy in mice; however, vector and promoter optimization for many cell types of the inner ear is still an active area of research [74]. Third, gene size is important due to the limited packaging capacity of AAVs (∼4.8 kb). While dual‐AAV approaches have enabled the delivery of larger genes (e.g., [40, 41]), triple‐AAV approaches remain experimental and have so far been less reliable [17]. Transgenes fitting into the single‐ or dual‐AAV capacity are currently preferred. Additionally, mouse models, and ideally additional models in other species (e.g., pigs and non‐human primates), recapitulating the human clinical situation are important for conducting studies to assess efficacy of preclinical therapies. Given the long‐term safety profiles of gene therapies remain unknown, the first trials will focus on severe or progressive hearing loss. Until safety and efficacy are better understood in human trials, it is likely that mild and moderate forms of hearing loss will not yet be ethically justified given risk–benefit considerations. Finally, the prevalence of each of the genetic forms of hearing loss influence the uptake to commercialization.

To illustrate the above: the most common form of hereditary hearing impairment is caused by *GJB2* (DFNB1A; OMIM: 121011), encoding Connexin 26, forming gap junctions among cochlear supporting cells and fibrocytes crucial for K^+^ recycling [79, 80]. Deleterious variants in *GJB2* in humans are associated with congenital severe to profound hearing impairment. At least half of patients initially present with residual hearing that is progressively lost [81]. The situation in the mouse is much different with homozygous deletion of *Gjb2* being lethal [79]. Conditional deletion of *Gjb2* in mouse models has been the only productive strategy to dissect cochlear pathology that shows disrupted cellular homeostasis early in development, with complete hair and supporting cell degradation before P30 [27]. Precise expression of *Gjb2* in correct cell types is crucial for integrity of electrical activity, but achieving this specificity with AAVs has been challenging. Most AAV vectors transduce multiple inner ear cell types and use strong, ubiquitous promoters, which can lead to mis‐expression. In the case of *Gjb2*, this has resulted in toxicity, including elevated hearing thresholds and lethality, highlighting the need for more targeted delivery strategies that are actively being pursued [24, 25, 26, 27]. Despite high human prevalence of *GJB2*‐associated hearing impairment and small gene size, several preclinical models have outlined a number of challenges for putative translation (Table 1).

## 4 *CABP2*: From Gene Discovery to Therapy Development

*CABP2* (OMIM: 607314), encoding Ca^2+^‐binding protein 2, was identified as causing autosomal recessive non‐syndromic hearing loss (DFNB93) through the identification of a founder variant (NM_016366.3:c.637+1G>T) in three consanguineous Iranian families presenting moderate to severe autosomal recessive hearing impairment with characteristic U‐shaped audiograms [82]. Follow‐up functional studies and detailed phenotyping of an Italian 4‐year‐old child with a homozygous loss‐of‐function variant (NM_016366.3:c.466G>T (p.Glu156Ter)) revealed mid‐frequency, moderate‐to‐severe hearing loss, and transitory evoked otoacoustic emissions [83]. This finding indicated preserved outer hair cell function, identifying the first individual with DFNB93 precisely diagnosed with an auditory synaptopathy.

**FIGURE 1:**
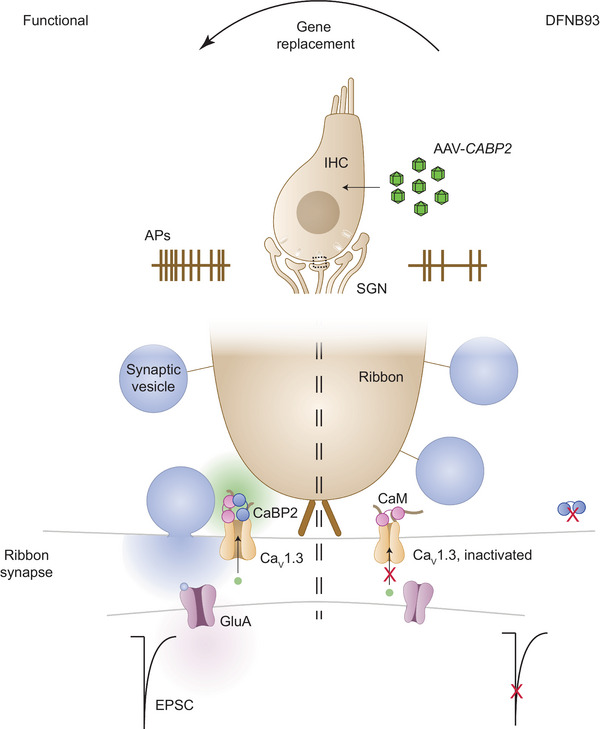
Function of CABP2 at the afferent synapse of inner hair cells (IHCs) and spiral ganglion neurons (SGNs) and gene replacement in DFNB93. CABP2 antagonizes voltage‐ and calmodulin (CaM)‐mediated inactivation of Ca_V_1.3 Ca^2+^ channels at the IHC active zones to sustain presynaptic glutamate release for indefatigable sound encoding. AAV‐mediated expression of transgenic *CABP2* restores Ca^2+^ influx and sound encoding in mouse models of DFNB93. Abbreviations: AP, action potential; EPSC, excitatory postsynaptic current; GluA, AMPA‐type glutamate receptor.

A mouse model with a deletion of *Cabp2* exons 3 and 4 showed reduced auditory brainstem responses and increased hearing thresholds despite preserved distortion product otoacoustic emissions corroborating auditory synaptopathy [83, 84]. The underlying synaptic disease mechanism is an enhanced steady‐state inactivation of inner hair cell (IHC) voltage‐gated Ca^2+^ channels due to the lack of CaBP2 regulation. This reduces the number of Ca^2+^ channels available for triggering glutamatergic transmission from IHCs impairing synaptic sound encoding by spiral ganglion neurons [16, 83] (Figure 1).

To date, nearly two dozen patients with *CABP2*‐associated hearing impairment have been published (Table 2), the majority of whom report moderate to severe hearing impairment. Measurement of otoacoustic emissions, so far, have been rarely done (Table 2), making comprehensive auditory phenotyping a high priority for further investigations. Replication studies to validate auditory synaptopathy are imperative. An additional point remaining poorly described in the literature concerns understanding speech in noise, which has important implications for the daily lives of affected individuals. In particular, mouse phenotyping indicates that auditory fatigue [16], that is, a pathological decline of the auditory percept during ongoing stimulation due to enhanced adaptation of auditory nerve spiking activity, will need to be assessed. Understanding how different *CABP2* variants contribute to phenotypic diversity and interfere with and modulate Ca^2+^ channel function remains to be explored in suitable disease models. Promising preclinical studies have yielded encouraging results [15, 16], showing the feasibility of *Cabp2* gene therapy, consolidating *CABP2* as an attractive target for therapy development. The first preclinical therapy of *Cabp2*‐associated hearing impairment used AAV2/1 and AAV‐PHP.eB vectors to deliver *Cabp2* coding sequence into the cochleae of P5‐7 *Cabp2^−/−^* mice [15]. High transduction efficiency and restoration of IHC Ca_V_1.3 function was reflected as improved hearing in *Cabp2^−/−^* mice. Further studies including *Cabp1* and *Cabp2* double knockout in the mouse model showed severely impaired IHC Ca^2+^ currents, synaptic transmission, and hearing that were all substantially recovered with transgenic expression of *Cabp2* [16].

**TABLE 2:** *CABP2* patients in the literature or clinical genetics databases.

Family ID	Ethnicity	Chr11 g. position (GRCh38)	*CABP2* c. position (NM_016366.3)/ (NM_001318496.2)	CABP2 p. position (NP_057450.2)/ (NP_001305425.1)	Zyg	Onset	Severity	TEOAE	Audiogram shape	Reference
Shf11	Iranian	g.67519792C>A	c.637+1G>T/ c.655+1G>T	p.?/ p.?	Hom	Prelingual	Moderate to severe	Negative	U‐shaped	[82]
Sh10	Iranian	g.67519792C>A	c.637+1G>T/ c.655+1G>T	p.?/ p.?	Hom	Prelingual	Moderate to severe	Negative	U‐shaped	[82]
He	Iranian	g.67519792C>A	c.637+1G>T/ c.655+1G>T	p.?/ p.?	Hom	Prelingual	Moderate to severe	Negative	U‐shaped	[82]
L‐1682	Iranian (Fars)	g.67519792C>A	c.637+1G>T/ c.655+1G>T	p.?/ p.?	Hom	Prelingual	Moderate to severe	NR	NR	[85]
L‐3201	Iranian (Fars)	g.67519792C>A	c.637+1G>T/ c.655+1G>T	p.?/ p.?	Hom	Prelingual	Moderate to severe	NR	NR	[85]
L8900091	Iranian (Turk)	g.67519792C>A	c.637+1G>T/ c.655+1G>T	p.?/ p.?	Hom	Prelingual	Moderate to severe	NR	NR	[85]
NA	Iranian	g.67519840A>G	c.590T>C/ c.608T>C	p.(Ile197Thr)/ p.(Ile203Thr)	Hom	Postlingual	Moderate to severe	NR	NR	[85]
NA	Iranian	g.67520074C>T	c.466G>A/ c.484G>A	p.(Glu156Lys)/ p.(Glu162Lys)	Hom	Prelingual	Moderate to severe	NR	NR	[85]
NA	European	g.67519792C>A	c.637+1G>T/ c.655+1G>T	p.?/ p.?	Hom	Prelingual	Moderate to profound	NR	NR	[86]
NA	Italian	g.67520074C>A	c.466G>T/ c.484G>T	p.(Glu156*)/ p.(Glu162*)	Hom	Prelingual	Moderate to severe	Initially present	U‐shaped	[83]
1239	Turkish	g.67519941C>A	c.490‐1G>T/ c.508‐1G>T	p.?/ p.?	Hom	NR	NR	NR	NR	[87]
DEM4545	Pakistani	g.67519792C>A	c.637+1G>T/ c.655+1G>T	p.?/ p.?	Hom	NR	NR	NR	NR	[88]
ISF‐13	Iranian	g.67521093C>T	c.311G>A/ c.329G>A	p.(Gly104Asp)/ p.(Gly110Asp)	Hom	Prelingual	Severe	NR	Sloping	[89]
DF13	Pakistani	g.67519792C>A	c.637+1G>T/ c.655+1G>T	p.?/ p.?	Hom	Prelingual	Moderate to severe	NR	U‐shaped	[90]
DF326	Jewish mixed ancestries (Syria/ Tunis, Turkey/ Ashk)	g.67521964C>T and g.67519792C>A	c.232G>A and c.655+1G>T/ c.250G>A and c.655+1G>T	p.(Glu78Lys) and p.?/ p.(Glu84Lys) and p.?	Comp het	Congenital	Moderate to profound	NR	U‐shaped	[91]
IPK4	Pakistani	g.67519792C>A	c.637+1G>T/ c.655+1G>T	p.?/ p.?	Hom	NR	NR	NR	U‐shaped	[92]
NSHL57	Danish	g.67519792C>A	c.637+1G>T/ c.655+1G>T	p.?/ p.?	Hom	Prelingual	Moderate	NR	U‐shaped	[93]
660	China	g.67520055del	c.487del / c.505del	p.(Glu163Serfs*55)/ p.(Glu169Serfs*55)	Hom	NR	NR	NR	NR	[94]
434	Puerto Rican	g.67519840A>G	c.590T>C/ c.608T>C	p.(Ile197Thr)/ p.(Ile203Thr)	Hom	Congenital	NR	NR	NR	[95]
Family A	Egyptian	g.67519840A>G	c.590T>C/ c.608T>C	p.(Ile197Thr)/ p.(Ile203Thr)	Hom	NR	Moderate to severe	NR	Ascending	[96]
Variation ID: 1698683	Iranian	g.67519948G>T	c.490‐8C>A/ c.508‐8C>A	p.?/ p.?	Hom	NR	Severe	NR	NR	ClinVar Accession: VCV001698683.2
Variation ID: 3340148	Brazilian	g.67521130G>A	c.274C>T/ c.292C>T	p.(Arg92*)/ p.(Arg98*)	Hom	NR	NR	NR	NR	ClinVar Accession: VCV003340148.1

*Note*: All individuals reported bilateral hearing impairment. The c. and p. position details are according to the human *CABP2* isoforms NM_016366.3 and NM_001318496.2, following the order indicated in the column headers.

Abbreviations: Comp het, compound heterozygous; Hom, homozygous; NA, not applicable; NR, not reported; TEOAE, transient‐evoked otoacoustic emissions; Zyg, zygosity.

Evaluation of known genes associated with isolated hearing loss yielded a short list that included *CABP2* among the best suited genes amenable for gene therapy, particularly due to preservation of cochlear architecture after P28, small gene size (human RNA size is 660 bp) allowing transgene packaging into a single AAV, and expression in hair cells [78]. The only unfavorable factor so far is the rarity of *CABP2*‐associated hearing impairment, which may be due to incomplete reporting and can thus be addressed with a patient registry.

## 5 The *CABP2* Registry: A Model for Precision Medicine in Rare Diseases

Given the rarity of *CABP2*‐associated hearing loss and its considerable therapeutic potential, we established the *CABP2* Registry (Clinical Trials Identifier: NCT06680934) as a centralized platform for patient data collection. It promotes collaboration among patients, who voluntarily contribute genetic and clinical data, and researchers, accelerating functional studies and discovery.

The registry utilizes a secure web‐based REDCap database. Participants complete the entire process online, from consent to data entry, using electronic case report forms. To ensure privacy, the system includes two separate databases: one for patient‐identifiable information and another for clinical and genetic data. Workflows, consent materials, and questionnaires were developed through a multidisciplinary collaboration among experts in otolaryngology, human genetics, and data protection and tested in both English and German. Study information materials and consent forms include versions for adults, parents, adolescents (14–17 years), and children (10–13 years), with parental co‐consent and electronic signature required for minors.

**FIGURE 2:**
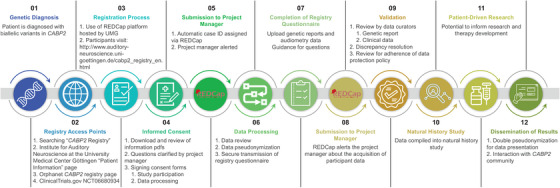
The *CABP2* Registry workflow and intended use of data to support clinical, basic, and translational research. The sequence of steps is numbered from left to right.

Figure 2 shows the registry workflow. Upon receiving a molecular genetic diagnosis of *CABP2*‐associated hearing impairment, participants can access study materials through multiple ways: a simple Google search for “*CABP2* Registry,” the Orphanet *CABP2* Registry page, ClinicalTrials.gov (NCT06680934), or the Institute for Auditory Neuroscience Patient Information page (http://www.auditory‐neuroscience.uni‐goettingen.de/cabp2_registry_en.html). Participants are encouraged to ask questions before providing digital consent. Upon enrolment, REDCap automatically generates a case identifier and notifies the project manager, who pseudonymizes the participant using an external key stored separately on a secure server. A unique registry access link is then emailed securely to the participant.

The registry questionnaire collects structured information about genetic test results, clinical course, family history, and hearing rehabilitation. Participants may upload supporting documents (e.g., audiograms and genetic reports) in a variety of possible file formats, provided that personal information is removed. Submissions are reviewed for completeness and compliance with data protection standards. Participants receive encrypted copies of their consent form and submitted data. They may revisit their personalized link any time to make corrections, update information, or add new audiometry. A second pseudonymization is applied prior to publication or presentation of data.

This registry consolidates key patient data, fosters collaboration, and accelerates research, particularly in the context of gene therapy development. One envisioned output is to support natural history studies by enabling long‐term tracking of disease progression and identifying potential interventional points. Given the limited commercial interest due to small patient populations, academic‐driven initiatives like this are essential for advancing new treatments. As genetic therapies progress, patient registries will play a key role in guiding translational research and improving care for individuals with hereditary hearing impairment. The *CABP2* Registry aims to connect patients who might otherwise face hurdles in participating in research studies, especially those in geographically dispersed or underrepresented areas, to ongoing research, providing a deeper understanding of disease mechanisms, therapy development, and clinical trial recruitment. Importantly, registries also empower patients to contribute to early‐stage research and the shaping of trial designs, and identify clinically meaningful endpoints. To our knowledge, this is the second registry for isolated hearing impairment that has been developed, following the success of the Otoferlin Registry [71]. As research for hearing restoration progresses, registries such as the *CABP2* Registry will play an increasingly important role to bridge translational application.

## 6 Next Steps for *CABP2* Therapy

The preclinical studies on *Cabp2^−/−^* models have shown potential for hearing restoration in DFNB93. However, continued efforts to further improve rescue of hearing in *Cabp2^−/−^* mice are warranted and should include optimization of AAVs and their dosages, timing of injection, as well as deeper research into promoter and enhancer sequences. One of the challenges of *CABP2* therapy, once optimized and ready for investigator‐initiated trials, will be access to patients. Identifying and selecting them based on motivation for therapy, severity, evidence of maintained otoacoustic emissions, and general eligibility will be important tasks. To proactively address this point, we have developed the *CABP2* Registry knowing that registries can be transformative yet underutilized tools. Therefore, we hope that this registry will increase odds of running clinical trials following further progress toward clinical trials.

## Author Contributions

Barbara Vona, Tina Pangršič, and Tobias Moser conceived the development of the registry. Barbara Vona, Bernd Wollnik, Nicola Strenzke, and Tobias Moser obtained study approvals and provided input during registry development. Barbara Vona wrote the first draft with input from all authors. All authors provided critical feedback, and reviewed and edited the manuscript.

## Ethics Statement

This study was approved by the Ethics Committee of the University Medical Center Göttingen (Approval: 17/8/22).

## Conflicts of Interest

Tobias Moser is an editorial board member of MedComm. He was not involved in the journal's review of or decisions related to this manuscript. The other authors declare no conflicts of interest.

## Data Availability

The authors have nothing to report.
